# Experimental Investigation of the Peel Strength of Artificial Leather and Polypropylene Specimens

**DOI:** 10.3390/polym15214217

**Published:** 2023-10-25

**Authors:** Deokrae Kim, Youngshin Kim, Euysik Jeon

**Affiliations:** 1 R&D Center, Yongsan Company, 62-48, Sinjeong-ro 293, Asan-si 31539, Republic of Korea; drkim@yong-san.com; 2 Department of Mechanical Engineering, Graduate School, Kongju National University, Cheonan-si 31080, Republic of Korea; 3 Graduate Program for Eco-Friendly Future Automotive Technology, Kongju National University, Cheonan-si 31080, Republic of Korea; 4 Industrial Technology Research Institute, Kongju National University, Cheonan-si 31080, Republic of Korea; 5 Department of Future Convergence Engineering, Kongju National University, Cheonan-si 31080, Republic of Korea

**Keywords:** polypropylene, surface treatment, artificial leather, peel strength

## Abstract

This study investigates the surface properties and adhesive strength of polypropylene (PP) in order to enhance the bond between PP injection-molded specimens and polyvinyl chloride (PVC) synthetic artificial leather. Plasma, primer, and flame treatments were applied to the surface of each specimen prepared using the two types of injection molds. The surface morphology, surface roughness, and contact angle were analyzed, and peel-strength analyses and a morphological inspections of the peeled specimens were performed. The peeling strength of the PP injection molding was measured, followed by a morphological examination of the peeled specimens. The plasma and flame treatments improved the peel strength, and the plasma and flame treatments changed the rough exterior to a hydrophilic surface, improving the peel strength. In addition, the primer treatment exhibited a lower peel strength than did the other treatments. This confirmed the low adhesion of the primer to the hydrophobic PP surface. The outcomes of this study can be employed across a multitude of industries that require improved adhesion for PP injection molded products.

## 1. Introduction

Polypropylene (PP) is widely used in injection molding owing to its excellent mechanical properties, affordability, and ease of processing. Injection-molded PP products are extensively used in the automotive, furniture, and fashion industries [1,2,3,4]. The demand for the attachment of fabrics to the surfaces of these products has grown, aiming to enhance the appearance of the product. Previously, natural leather was commonly used for this purpose; however, environmental concerns have led to the adoption of artificial leather. Artificial leather, such as polyurethane (PU) leather and polyvinyl chloride (PVC) leather, is manufactured to replicate the texture and feel of natural leather [5,6,7]. By coating, dipping, laminating, and other processes, PU and PVC materials are applied to the artificial leather, followed by buffing or polishing to create a leather-like pattern and texture. The flexibility and durability of artificial leather make it suitable for various industries, allowing it to be applied to the surface of injection-molded parts, expanding their range of applications [8,9,10].

However, owing to the nonpolar nature of PP, causing poor wetting and adhesion [11,12], PP surfaces exhibit low surface energy, which hinders the formation of strong adhesive bonds [13]. To address this issue, several methods have been explored to improve the adhesion of PP materials. Surface treatment techniques, such as plasma treatment and chemical etching, have been proposed to increase the surface energy, thereby enhancing wettability [14,15,16,17]. Modifying the surface characteristics to improve adhesive receptivity and changing the surface roughness of PP through mechanical engagement are additional approaches for improving adhesive bonding strength [18].

Many researchers have explored the impact of plasma treatment on the bond strength of PP materials. For instance, Navaneetha et al. [19] investigated the adhesion strength characteristics of PP materials by treating PP and polyethylene terephthalate (PET) surfaces with direct-current (DC) glow discharge plasma. Mandolfino [20] examined the changes in plasma characteristics based on process time, gas type, and voltage. Plasma treatment is widely used to achieve adhesion strength and surface treatment across various fields, not limited to PP materials. Furthermore, Tomoya et al. [21] used the flame treatment method to analyze the adhesion strength characteristics on the surface of PP specimens [22,23]. The adhesion strength between PP materials and artificial leather has been investigated by examining the interfacial bond characteristics between the PP fibers and a concrete matrix. Factors such as hybrid fiber content, matrix strength, and embedded fiber length have been considered to analyze the frictional bond strength of PP fibers. Furthermore, studies have been conducted to improve the adhesion strength of PP materials to steel, hybrid fiber reinforced concrete, and cement matrix for use as construction materials [24,25]. However, most of these studies focused primarily on analyzing the individual effects of surface treatment techniques, and research regarding surface condition and bond-strength characterization remains scarce.

In this study, we examined the surface properties and adhesion strength characteristics of injection molded PP and PVC artificial leather. We aimed to improve the adhesion strength by analyzing the surface condition and treatment methods. Two types of injection molds were utilized to investigate how surface roughness affects adhesion strength in PP injection parts: one with etching post-milling, and the other without any additional treatment, enabling the injection of specimens with varying surface roughness. This study represents a significant advancement when compared with previous research, as it enables a simultaneous comparison of surface roughness, wettability, and adhesion strength characteristics for different treatment methods. Such comprehensive analysis is highly valuable in various industrial fields that seek to enhance the adhesion of PP injection parts.

## 2. Surface Treatments and Characterizations

### 2.1. Materials

In this study, PP (PP-(MH + TD)10, GS Caltex Co., Ltd., Seoul, Republic of Korea) was used as the research target. According to the PP Technical Data Sheet, the material exhibits a tensile strength of 18 MPa, an elongation percentage of 180%, and a flexural strength of 27 MPa. The Izod impact strength at 23 °C is 41 kJ/m^2^. The material has a melt flow index of 11 and a specific gravity of 0.95. The supplied PP material is a PP resin with fillers such as tar, giving the specimen a black color. In the adhesive experiments and tests involving the PP samples, PVC artificial leather, with woven fibers as the base material, was employed. The PVC artificial leather was provided by I&S Co., Ltd., Cheongju, Republic of Korea, and manufactured using the casting method. In this method, a paste resin in a solution state was coated on a release paper, and a binder was laminated to produce sheets or fabrics [26,27,28]. PVC artificial leather exhibited a precise surface texture, patterns, excellent durability, and abrasion resistance [5,10]. The PVC artificial leather was 1.05 mm thick, with a tensile strength of 117 N/30 mm, a percent elongation of 120%, and a tear strength of 14.7 N. The PVC artificial leather was cut to the size required for the peel-strength test (160 mm × 50 mm).

To enhance the adhesive strength of PP for automotive interior bonding applications, a primer containing SikaSense’s chlorinated polyolefin compound (Part number 4670P, Sika, Baar, Switzerland) was applied. The components of the primer were methylcyclohexane (C_7_H_14_) and p-tert-butylphenyl 1-(2,3-epoxy)propyl ether (C_13_H_18_O_2_). In addition, a PU-based water adhesive SikaTherm^®®^-4250 (Sika, Baar, Switzerland) was used to bond the PP specimen to the leather. SikaTherm^®®^-4250 is composed of 5-chloro-2-methyl-4-isothiazolin-3-one and 2-methyl-2H-isothiazol-3-one.

### 2.2. Specimens Preparation and Surface Treatment

The specimens were produced using a DEIA250 injection molding machine (HYUNDAI INJECTION MACHINERY Co., Ltd., Foshan, China) with a clamping force of 250 tons. The injection conditions included melt temperatures ranging from 195 °C to 210 °C for each section, injection pressures ranging from 45 MPa to 55 MPa for each section, and a cycle time of 50 s. The mold temperature was set to 50 °C. To prevent the difference in peel strength caused by the release agent on the surface of the specimen, the specimen was injected without into the mold any release agent. The size of the injection specimen was 300 mm × 100 mm.

The surface of the injection parts was significantly influenced by the injection mold surface during the injection molding process [5,29]. For this analysis, two types of injection molds were employed to examine the adhesion characteristics of the PP specimen surface, based on their shape. The first mold underwent no post-processing after milling, whereas the second mold was post-processed by attaching a patterned film to the mold and etching it to create a fine mesh pattern on the surface of the mold.

**Figure 1:**
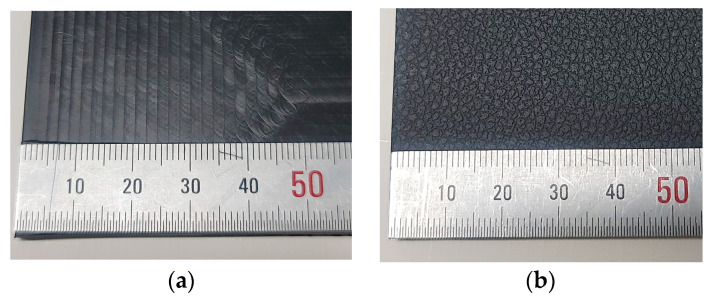
PP-injection specimens: (**a**) PP specimen surface with non-etched mold (PP); (**b**) PP specimen surface with etched mold (PPm).

Figure 1 shows the actual surface-condition image of the PP specimen. The specimens injected using the first mold displayed linear milling marks on the surface (Figure 1a), whereas those injected using the etching mold exhibited a dot pattern (Figure 1b). Three surface treatments were performed on both types of PP-injection specimens: plasma treatment, known as a common surface treatment method for polymer materials; flame treatment; and primer application. The reference specimen did not undergo any surface treatment. The names assigned to the specimens based on the mold conditions and the surface-treatment conditions are listed in Table 1. In the case of PP specimens without etching on the mold surface, the specimen name started with PP, and in the case of PP specimens that were injected after etching on the mold surface, the specimen name was PPm. In addition, the specimen name was created to identify the surface-treatment method so that it could be distinguished through the specimen name. The injected specimens were cut into the selected peel-strength-specimen size (160 mm × 50 mm), and surface treatment was performed. The specimens for surface treatment were prepared with 10 specimens per condition for surface analysis and peel strength measurement.

In this study, air plasma was used for treating the surface of the PP specimens, employing the AP-4000 model plasma device (AETP, Co., Ltd., Donghae, Republic of Korea). The treatment involved applying 1000 W of power, maintaining a 10 mm distance between the plasma nozzle and the specimen, and moving at a traverse speed of 250 mm/s. After plasma surface treatment, surface characterization and specimen preparation were performed within 1 h after plasma treatment. Tests were performed as soon as possible after surface treatment to avoid the influence of aging variation [21]. For flame treatment (AFP-250T model, AETP, Co., Ltd., Donghae, Republic of Korea), a mixture of 200 L/min of air and 17 L/min of liquefied petroleum gas was used, with a 70 mm distance between the specimen and the nozzle. The flame-treatment speed was set to 500 mm/s. The primer was coated on the specimens with a weight of 153 g/m^2^. The primer application device used was a versatile coating robot (EPX2050, Yaskawa Electric Corporation, Kitakyushu, Japan). After a five-minute drying period at room temperature (25 °C), further drying at 45 °C was conducted for 10 min.

To bond PP specimens with the PVC artificial leather after surface treatment, the adhesive was applied using the spray method at a pressure of 7 bar, and 260 g/m^2^ of adhesive was applied to each specimen. The specimens were allowed to dry at room temperature (25 °C) for 30 min before further usage. The curing of the adhesive was achieved by applying a mixture of the water-based adhesive and the curing agent at a certain ratio (100:6), and the curing occurred by drying, owing to the evaporation of the solvent and the chemical reaction of the curing agent.

**Figure 2:**
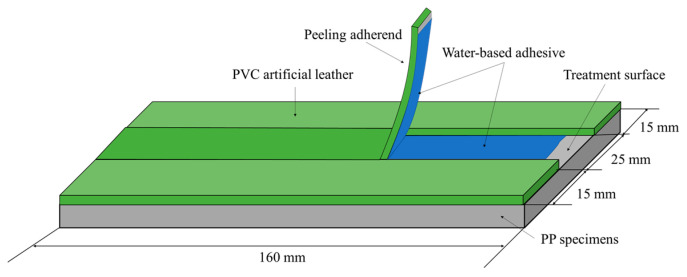
Conceptual diagram of a specimen for the peel-strength experiment.

After applying the adhesive to the PP specimens and PVC artificial leather, the specimens were bonded by pressing at a pressure of 500 kPa for 1 min, and the temperature during bonding was set to 65 °C, where the water-based bond exerts adhesion. Three copies of each specimen were prepared for repeated peeling-strength experiments. Figure 2 shows the dimensions and a conceptual drawing of the specimen for the peel strength test.

### 2.3. Characterization of Samples

**Figure 3:**
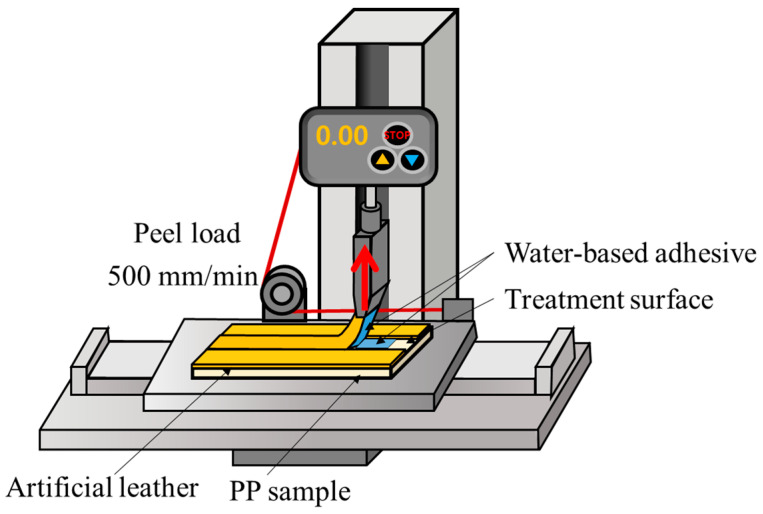
Conceptual diagram of the experimental specimens and peel-strength experiments.

Scanning electron microscopy (SEM) was performed using a Carl-Zeiss Sigma 500 microscope (ZEISS, Oberkochen, Germany), with an acceleration voltage of 10 kV. A platinum coating was applied under vacuum conditions. The apparent contact angle of the PP specimen was measured using a contact-angle analyzer (Phoenix 300 Touch, SEO Co., Ltd., Suwon, Republic of Korea) based on the sessile drop method. Apparent contact-angle measurements were performed at a room temperature of 22 °C and a humidity of 53%, with water as the experimental solvent. Measurements were performed using the static contact-angle method [30]. In this test method, drops of water are placed on the surface of a sample to indicate the metastable state of a liquid drop on the sample. In general, the apparent contact angle has a random value because the metastable state of two successive droplets may be different [31,32]. The value of the apparent contact angle within the range of contact-angle hysteresis is limited by advancing and receding contact angles [33]. The droplet volume for the contact-angle measurements was set to 7 µL. The droplet volume should not be too small, as small droplets are susceptible to vibration, evaporation, and optical errors. The larger a droplet, the more gravity distorts its shape. The typical droplet volume range for the sessile drop method is 3–20 µL [31,33]. The drops were dropped from a height of 1 mm, and the contact angle was measured within 2 s of dropping the liquid onto the surface. Contact-angle measurements were repeated five times per specimen. When the measurements were repeated five times for each sample, the sample was moved and measured so that the water droplets did not overlap. The contact angle was calculated by measuring both the right and left angles of the drop and averaging them. Digital images of the liquid-droplet shape were captured for the contact-angle calculations. Atomic force microscopy (AFM) in the non-contact mode was employed to assess the surface roughness of the PP specimens. Surface images were obtained over a 20 × 20 μm^2^ area using a Park NX10 AFM (Park Systems Corp., Suwon, Republic of Korea). The adhesive strength between the PP specimens and artificial leather was measured according to the ISO 813 standard, which outlines a method for evaluating the adhesive strength between flexible rubber materials and rigid substrates. The peel strength was measured using a UTM SHMF2-C-Series (SAMHAN Technology, Bucheon, Republic of Korea) device. A 50 mm section at the end of the substrate was forcefully peeled, and the specimen was secured on a jig plate using tension clamps. A 25 mm wide test specimen underwent a 90° angle peel test at a speed of 500 mm/min to determine the maximum test load. The peel-strength test was performed at room temperature (25 °C) on three replicates of the specimens, prepared according to the surface treatment method. Figure 3 presents a schematic of the conducted peel-test experiments. The cross-sections were cut from the specimens, and the peeled surfaces were examined using SEM to analyze their morphologies.

## 3. Results and Discussion

### 3.1. Morphologies

**Figure 4:**
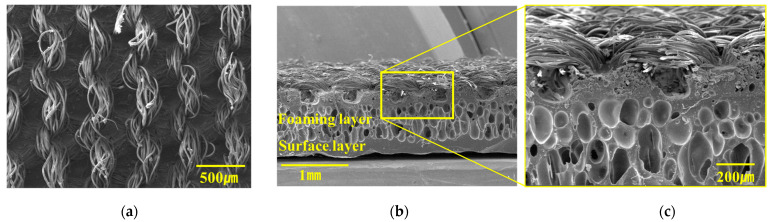
Surface morphology of the PVC artificial leather: (**a**) surface of PVC artificial leather adhering to the PP specimen; (**b**) PVC cross-section; and (**c**) enlarged image of a PVC cross-section.

SEM measurements were conducted to assess the surface condition of the synthetic-fiber artificial leather; the results are shown in Figure 4. Figure 4a displays an image of the PVC artificial leather surface intended for bonding, revealing bundled fibers with a twisting pattern. Figure 4b,c provides cross-sections of the PVC artificial leather, illustrating the pore shapes formed during solution coating and binder application. These patterns indicate the cross-sectional shape of the leatherette [34].

**Figure 5:**
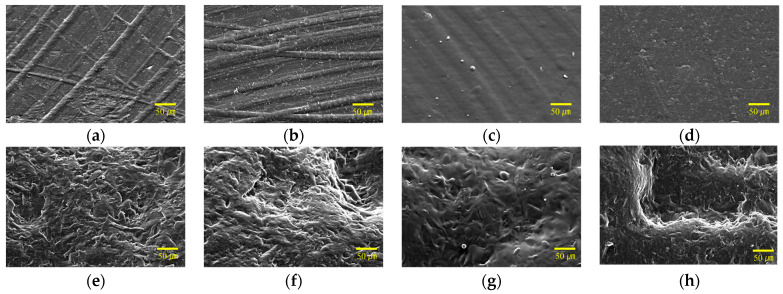
SEM images of PP injection specimens: (**a**) PP-Ref, (**b**) PP-Pl, (**c**) PP-Pr, (**d**) PP-Fl, (**e**) PPm-Ref, (**f**) PPm-Pl, (**g**) PPm-Pr, and (**h**) PPm-Fl.

The surface condition of the PP injection specimens was examined using SEM measurements. Figure 5 shows the surface measurements of the plasma-treated, primer-applied, and flame-treated specimens. In the case of the untreated mold specimen (Figure 5a), streaks caused by injection mold processing appear as machining marks on the injection specimen. Figure 5e displays an uneven pattern resulting from the surface-etching treatment geometry of the injection mold. When comparing the surfaces of the reference and plasma-treated specimens (Figure 5b,f), no significant differences were observed. Plasma treatment primarily impacts the chemical reactivity, without altering the physical-surface morphology. After primer application and flame treatment, the surface features appear dull and blurry, compared with the untreated reference specimen [35]. Figure 5c demonstrates that the pattern of thin and long lines caused by the mold machining marks becomes less distinct, and the applied primer gives it a smoother appearance (Figure 5g). In the case of flame treatment (Figure 5d,h), the surface curvature appears melted and flattened. For the molded specimen, the irregularly shaped bends are reduced and smoothed out with primer application, whereas flame treatment reduces the small patterns, leaving only the overall large pattern.

### 3.2. Analysis of the Contact Angle of PP Specimens

**Figure 6:**
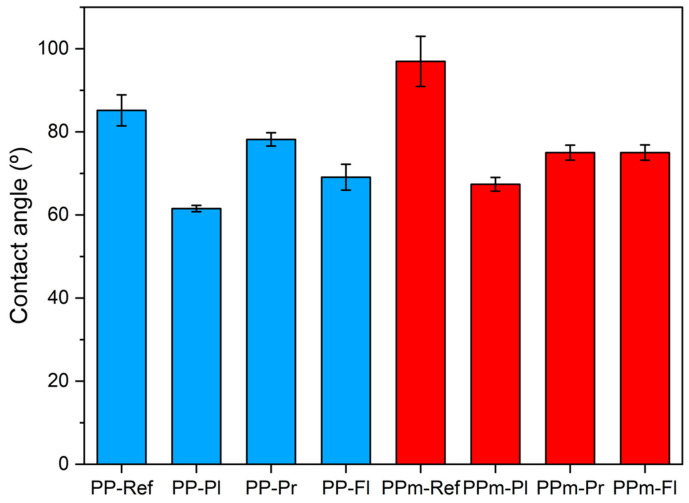
Results of the contact angle by surface treatment.

**Figure 7:**
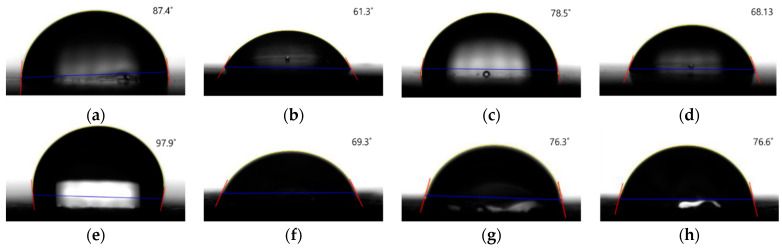
Contact-angle-measurement images of PP-injection specimens.(The blue line is the baseline, and the red line is for measuring the angle of the droplet): (**a**) PP-Ref, (**b**) PP-Pl, (**c**) PP-Pr, (**d**) PP-Fl, (**e**) PPm-Ref, (**f**) PPm-Pl, (**g**) PPm-Pr, and (**h**) PPm-Fl.

Figure 6 displays the apparent contact-angle-measurement results for each surface-treatment condition. The graph shows the deviation and average of the repeated measurements. The blue bars represent the contact angles for PP specimens injected with an unetched mold under different surface-treatment conditions (PP-Ref, PP-Pl, PP-Pr, and PP-Fl). The red bars represent the contact angles for specimens injected with PP using an etched mold under different surface-treatment conditions (PPm-Ref, PPm-Pl, PPm-Pr, and PPm-Fl). For the PP specimens injected with an unetched mold, the contact angle decreases for all treatments. Plasma treatment causes the most significant change in the contact angle. Figure 7a shows the reference specimen with a contact angle of 87.4° before surface treatment, which is close to 90°. Notably, the contact angle significantly reduces after plasma treatment (Figure 7b, PP-Pl contact angle: 61°), primer application (Figure 7c, PP-Pr contact angle: 78°), and flame treatment (Figure 7d, PP-Fl contact angle: 68°). The decrease in the contact angle is attributed to the plasma treatment, resulting in the formation of free radicals on the polymer surface and promoting interaction with water molecules to overcome surface tension [36,37]. Flame treatment and primer application also led to improved hydrophilicity. Similar results were observed for specimens produced by the etched mold. The reference specimen with the etched mold (Figure 7e) exhibits a large contact angle, with an average of 97.8°, which decreases with plasma treatment (Figure 7f). Contact angles also reduce for PPm-Pr (Figure 7g) and PPm-Fl (Figure 7h) specimens. Although the extent of change varies depending on the surface-treatment method, the contact angle decreases in all cases. This reduction indicates an improved wettability and provides an opportunity to enhance the adhesive strength [38,39].

**Figure 8:**
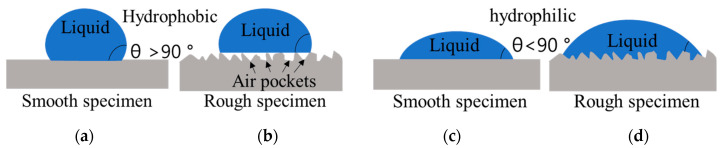
Schematics of the contact angle according to surface roughness and hydrophobicity: (**a**) smooth surface and hydrophobic specimen; (**b**) rough surface and hydrophobic specimen (Cassie–Baxter state); (**c**) smooth surface and hydrophilic specimen; (**d**) rough surface and hydrophilic specimen (Wenzel state).

Figure 8 shows the change in the contact angle as a function of the surface condition and wettability. The Young equation is the basic relationship linking the wetting properties of the liquid with the contact angle [40]. Young Equation (1) describes how the contact angle (*θ*) at the three-phase boundary of a liquid droplet on a solid surface is determined by the interfacial free energies: the interfacial free energy between the liquid and vapor ($\gamma_{L}$), the solid and gas ($\gamma_{s}$), and the solid and liquid ($\gamma_{sL}$).

(1) $$\gamma_{L}cos\theta =\gamma_{s}-\gamma_{sL}$$

In practical terms, the Young equation is applicable to idealized situations involving perfectly smooth, chemically uniform, nonreactive, and rigid surfaces in an equilibrium state. However, real-world surfaces often possess imperfections and variations in both their geometry and chemical properties. Young’s equation cannot be verified experimentally because the solid surface energies $\gamma_{s}$ and $\gamma_{sL}$ cannot be measured independently (only changes in the solid surface energies can be measured) [41]. When dealing with surfaces that are heterogeneous, rough, or have a complex structure, it becomes valuable to introduce the concept of apparent contact angles. Apparent contact angles are established based on the empirical observations of liquid droplet interactions with surfaces, with due consideration of the surface’s inherent irregularities and chemical heterogeneities. These apparent contact angles offer a pragmatic approach for characterizing the interactions between liquids and real-world surfaces that diverge from the idealized assumptions inherent in Young’s equation. They comprehensively account for the collective impact of surface topography and the nuanced variations in surface energy, thereby enhancing our comprehension of wetting phenomena and the dynamics of interactions in intricate systems [32,42]. An apparent contact angle is defined when we consider observations at a scale length significantly larger than the surface’s structural features or chemical variations. In essence, it denotes the macroscopic contact angle that becomes apparent when we adopt a broader perspective and choose to overlook the intricate specifics related to surface roughness or chemical variances. This concept serves as a simplification strategy in characterizing wetting phenomena on intricate surfaces, allowing us to concentrate on the comprehensive, observable behavior occurring on a more extensive scale [43].

The change in the apparent contact angle resulting from surface roughness can be observed by comparing the contact angles of PP-Ref and PPm-Ref; the apparent contact angle of PPm-Ref is larger owing to the large contact angle caused by air pockets generated on the surface [44,45]. This phenomenon causes a larger contact angle on hydrophobic surfaces. For simplicity, two basic wetting states, the Wenzel and Cassie–Baxter states, are considered when liquid wets a rough surface (Figure 8b,d) [44,46]. In general, hydrophobic surfaces have contact angles (*θ*) greater than initial 90° (Figure 8a,b) [47,48]. The advancing contact line can pin at various asperities on the rough surface, resulting in an anomalously large contact angle. The apparent contact angle can be reduced by modifying the surface to become hydrophilic through various surface treatments, such as plasma treatment, on the specimen surface (Figure 8c,d). Liquid fully wets the entire area of the rough surface. For a hydrophilic material, the wettability is enhanced, rendering the surface highly hydrophilic. Under such conditions, both advancing and receding angles are small; thus, so is the hysteresis. This surface modification is known to improve the bond strength [49].

### 3.3. Analysis of PP Specimen Roughness

**Figure 9:**
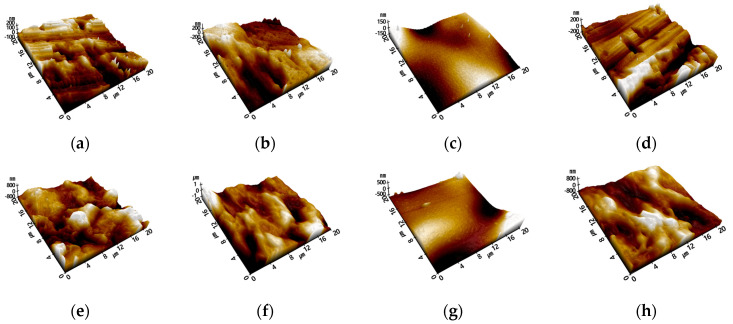
SEM measurement images of PP injection specimens; (**a**) PP-Ref, (**b**) PP-Pl, (**c**) PP-Pr, (**d**) PP-Fl, (**e**) PPm-Ref, (**f**) PPm-Pl, (**g**) PPm-Pr, and (**h**) PPm-Fl.

The results of measuring the condition of PP injection specimens with different surface-treatment conditions are shown in Figure 9. The surface condition varies, depending on whether the mold is etched or not. The linear pattern observed in Figure 9a aligns with previous SEM measurement results, and the irregular embossing pattern is evident in Figure 9e. Figure 9b,f displays some blunting of the features due to plasma treatment, whereas Figure 9c,g shows the primer covering the mold patterns, resulting in a dull surface. Additionally, the surface after flame treatment (Figure 9d,h) exhibits a similar shape to that of the untreated reference specimen.

Table 2 lists the surface-roughness parameters measured by AFM. Rpv is the maximum-height difference of the peak–valley structure, and Rq is the surface root-mean-square roughness. Ra indicates the sum of all absolute values of height based on the mean line and divided by the line length, and Rz denotes the average of the absolute values of the five highest and five lowest points based on the mean line. Rsk denotes the up–down asymmetry relative to the mean line, with a value of 0 indicating symmetry, greater than 0 indicating a downward skew, and less than 0 indicating an upward skew. Rku is a measure of the height distribution relative to the mean line, with values greater than 3 showing a steeper distribution than the normal, and values less than 3 exhibiting a flatter distribution than the normal. The data in Table 2 present the mean values and standard deviations of the experimental results of three replicates for each specimen for the surface roughness parameter. The measurement position was selected as the center of the specimen and was measured relative to the center point within the measurement area [50,51,52]. Compared with the PP-Ref specimen, overall, the surface-roughness parameters of the PPm-Ref specimen exhibited larger values, and Rsk was measured as a negative value for both specimens. Compared with the PP-Ref specimen, overall, the surface-roughness parameters of the PPm-Ref specimen exhibited larger values, and Rsk was measured as a negative value for both specimens. Rku values exhibited relatively higher values for the PP-Ref specimen. These values exhibited changes owing to the surface treatment. It can be observed that the height-related parameters Rpv, Rq, Ra, and Rz are all increased for the PP-Pl and PP-Fl specimens compared with the PP-Ref. This confirms the increase in surface roughness due to the surface treatment. However, for PP-Pr, the height-related parameters Rpv, Rq, Ra, and Rz all decreased compared with those of the PP-Ref. In addition, the Rsk values all increased and the Rku values decreased compared with those of the reference specimen. For the PPm-Pl, PPm-Pr, and PPm-Fl specimens, most values decreased compared with those of the PPm-Ref, and some PPm-Pl values increased, but they remained within the error range. In addition, both Rsk and Rku increased compared with the PPm-Ref specimens.

### 3.4. Bonding Strength of PP-Skin

**Figure 10:**
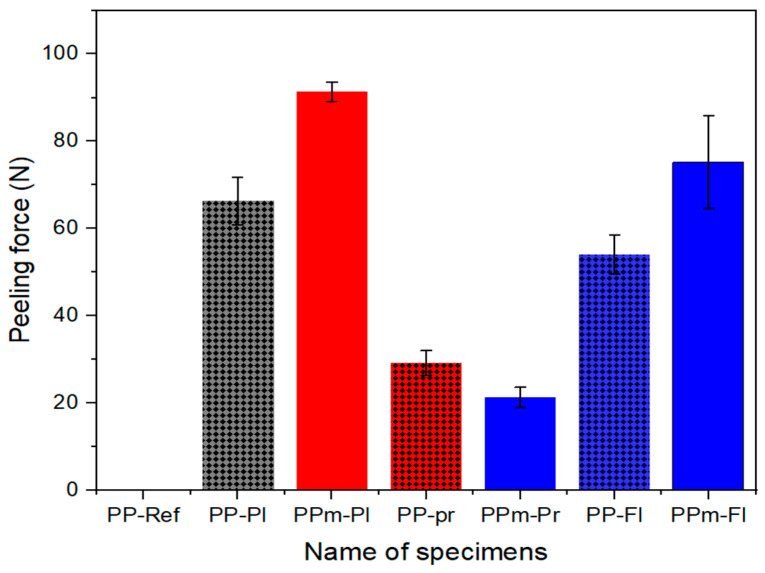
Peel strength test results by surface condition and surface treatment.

**Figure A1:**
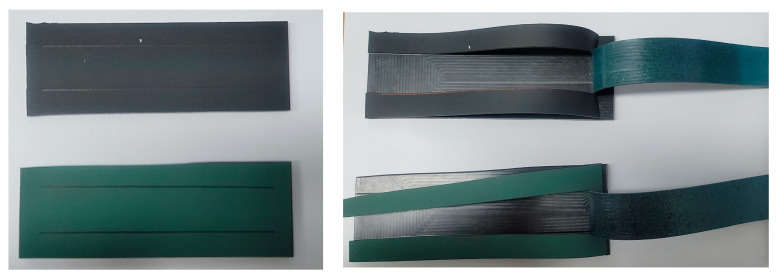
Peeling-strength test of untreated PP specimens and PVC artificial leather. The water-based adhesive applied to the PP surface came off, even without applying any force.

The experimental results of the peel strength of the PP specimen and PVC artificial leather are shown in Figure 10. Before the surface treatments, the PP showed no adhesive properties, as shown in Figure A1 in Appendix A. The solid bars represent the peel strength of the specimens injected using the non-etched mold, whereas the solid bars with black dots represent specimens injected using the etched mold. For the PP specimens treated with plasma and flame, a higher peel strength is observed when they were injected using the etched mold. The adhesion strength of the PPm-Pl and PPm-Fl specimens improved by 38% and 39% compared with that of PP-Pl and PP-Fl, respectively. The specimens extruded from the etched mold exhibited increased surface roughness, leading to a larger contact-surface area for the artificial leather. This increased contact area provides ager bonding surface and improves the overall strength of the adhesive bond [53]. Rough and hydrophilic surfaces possess irregularities such as peaks, valleys, and micro-scale features. These surface characteristics allow adhesives to flow into the irregularities, creating mechanical interlocking and enhancing adhesive strength. The interlocking effect becomes stronger with more pronounced surface roughness. Additionally, rough surfaces generate higher frictional forces between the adhesive and the substrate, resisting separation forces and preventing premature detachment. The increased frictional resistance contributes to improved adhesive strength [54,55]. Plasma and flame treatments generally improve bond strength. However, the primer-treated specimens exhibited very low peel strengths compared with those of the other surface treatments. The PPm-Pr specimen exhibited a more than 70% reduction in peel strength compared with the PPm-Pl specimen, which has the highest peel strength, and the PP-Pr specimen exhibited a 56% reduction in peel strength compared with the PP-Pl specimen. The reason for these results was confirmed by observing the shapes of the delaminated specimens.

**Figure 11:**
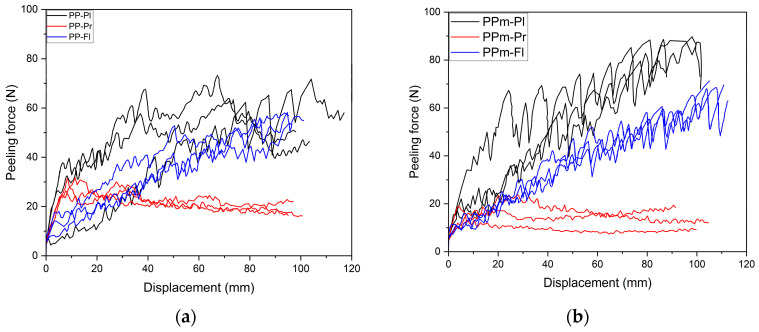
Peel-strength experimental results for the surface condition and surface-treatment method: (**a**) peeling force and displacement of non-etched mold specimens; (**b**) peeling force and displacement of etched mold specimens.

The results of the peel-strength test, including peeling load and strain for the PP specimen and PVC artificial leather are shown in Figure 11. The graph shows the results for all three replicates. The black solid line shows the peel strength of the plasma-treated specimen, the blue solid line shows the results of the flame-treated specimen, and the red line shows the results of the primer-treated specimen. Figure 11a displays the peeling-test results for specimens injected through the mold without etching treatment, and Figure 11b shows the peeling strength for specimens injected through the etched mold. In the case of primer application, the peel strength is initially higher and then slightly decreases during the peel test. These results were confirmed by a geometric analysis of the specimens after peeling. Peeling occurs at a similar load, and consistent with previous findings, the peel strength of the PP specimen from the mold without etching treatment is higher.

For plasma- and flame-treated specimens, the peel strength of specimens injected through the etched mold tends to fluctuate, with alternating increases and decreases. As discussed earlier in the analysis of maximum peel strength, the specimens injected through the etched mold exhibited higher peel strength.

### 3.5. Analysis of Delamination of PP-Skin

**Figure 12:**
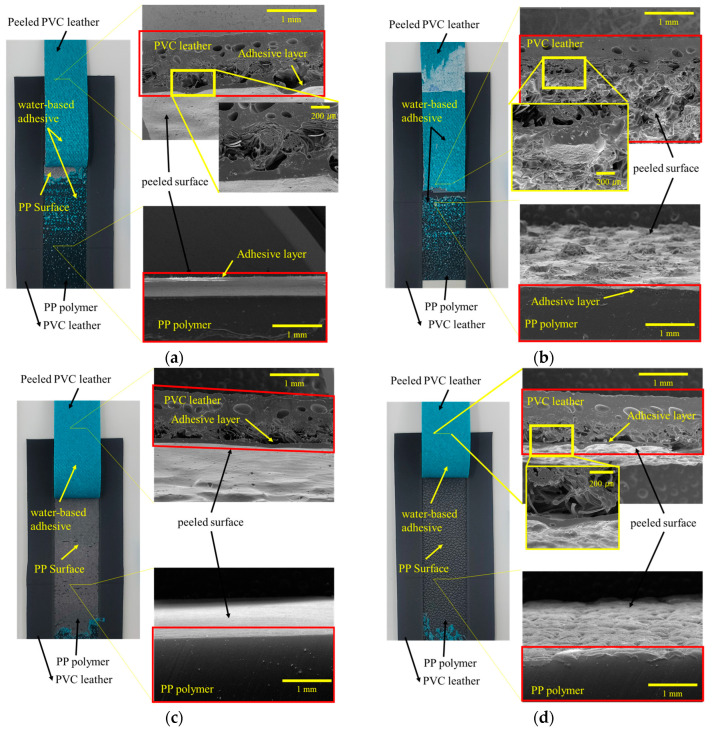

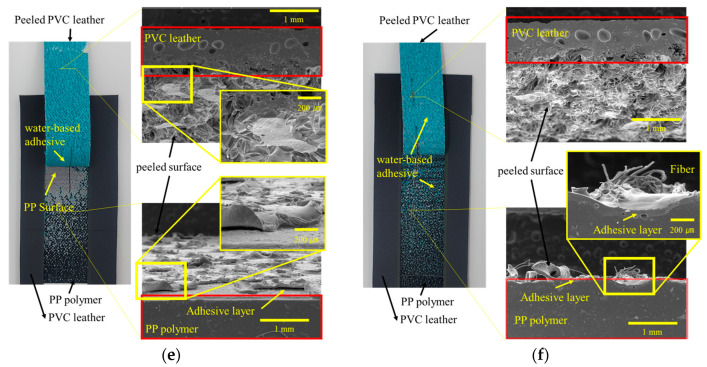
SEM and photographs of the fracture morphology after the peeling test: (**a**) PP-PL, (**b**) PPm-PL, (**c**) PP-Pr, (**d**) PPm-Pr, (**e**) PP-FL, and (**f**) PPm-FL.

To examine the failure morphology of fractured specimens after the peel-strength test, SEM analysis was conducted on the PP specimens and the artificial leather. Specific locations were selected and analyzed to characterize different types of delamination. Figure 12 illustrates the SEM measurement results and photographs of the specimens after the peel test. The image presented in Figure 12 is a photograph captured after the peeling experiment, and the blue part of the photograph is the applied water-based bond; the peeling form of the water-based bond varies, depending on the surface treatment method. SEM sections of the peeled artificial leather and PP specimens are shown in red boxes. Figure 12a shows the results of the peel test conducted on the PVC leather after plasma treatment of PP specimens injected through an unetched mold. The initial peel strength is relatively low owing to the separation of the bond between the PVC artificial leather and the PP specimen. Figure 12b displays the results of the peel test on the PVC artificial leather after plasma treatment of the PP specimens injected through an etched mold. The highest peel strength is observed at the end of the test as the artificial leather is torn, with the bond layer applied to the leather surface peeling off toward the PP side. The fibers on the PVC artificial-leather surface appear stretched, indicating some separation. Figure 12c,d depicts the peeling pattern of the specimens after the peel test, following primer treatment. In both cases, the primer layer delaminates from the PP specimen and moves toward the artificial leather. Notably, for the primer-treated specimen, lower adhesion strength is observed with the PP specimen injected using the etched mold. This is attributed to the smaller contact area between the primer and PP specimen owing to surface roughness and the presence of air pockets, resulting in reduced peel strength. Figure 12e,f presents the peel-test results for the flame-treated specimens, showing a similar peeling morphology as that observed with plasma treatment. The applied bonds on the PVC surface migrate toward the PP specimen.

**Figure 13:**
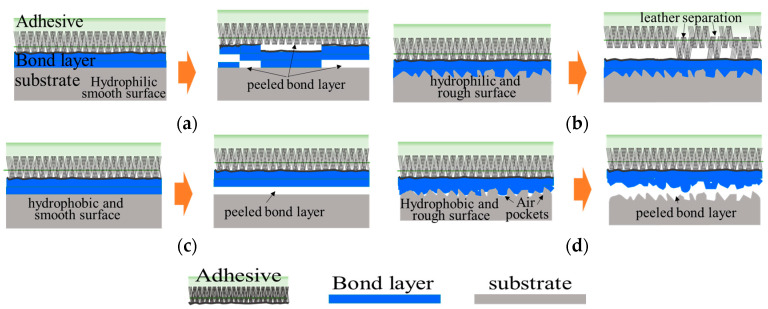
Conceptual illustration of adhesive application and peeling geometry according to the surface roughness and hydrophilicity of the specimen: (**a**) conceptual illustration of adhesion and failure on smooth and hydrophilic surfaces, (**b**) conceptual illustration of adhesion and failure on rough and hydrophilic surfaces, (**c**) conceptual illustration of adhesion and failure on smooth and hydrophobic surfaces, and (**d**) conceptual illustration of adhesion and failure on rough and hydrophobic surfaces.

The delamination experiment revealed different types of delamination. Figure 13 displays the peeling morphology, where Figure 13a,b illustrates the adhesive application and the peeling shapes on surfaces treated to develop hydrophilic properties. Figure 13a,b depict evenly distributed adhesives on the hydrophilic surfaces, with varying surface roughness. In such cases, higher surface roughness during peeling leads to stronger adhesion and bonding force due to artificial-leather separation. This characteristic is also observed for the plasma and flame treatments, resulting in high peel strength. However, Figure 13c,d demonstrates adhesive application and peeling on hydrophobic surfaces. Notably, hydrophobicity with a rough surface leads to decreased peeling strength due to adhesive peeling from voids between the specimen and the adhesive, as observed when the primer was applied. The untreated specimen with a rough surface is hydrophobic, with a contact angle of more than 90°, and when a primer is applied to this surface, peeling occurs between the rough surface of the PP and the primer, reducing the bonding strength. On hydrophobic surfaces, the presence of air pockets and limited adhesive contact with certain areas reduces the length of contact with the surface [56]. To overcome this effect, is it is necessary to increase the hydrophilic characteristics. This modification enhances the length of the adhesive interface and mechanical interlocking, amplifying the impact of surface roughness.

## 4. Conclusions

In this study, we analyzed the surface properties and adhesion-strength characteristics of artificial leathers to enhance the bond between PP injection molded products and PVC artificial leathers. Plasma, primer, and flame treatments were performed on the surface of each specimen, and the peel strength was analyzed according to the surface condition and surface-treatment method. We confirmed that plasma and flame treatment methods improve the adhesion strength of PP and PVC; furthermore, our findings revealed that surface roughness and the hydrophilic surface of the PP material significantly impact the adhesion strength. Applying plasma and flame treatments to the PP surface increased adhesion strength, especially for specimens with rougher surfaces. This can be attributed to the interlocking mechanism, whereby surface treatment enhances the adhesive hydrophilicity and contact length through increased surface roughness. However, for primer-coated specimens, we observed a low adhesive strength for rougher surfaces. This is attributed to the hydrophobic and rough surface of the PP surface causing air pockets between the primer and the PP surface. These findings have practical applications in various industrial sectors that require improved adhesion for PP injection parts.

## Figures and Tables

**Table 1:** Names of specimens based on mold conditions and surface-treatment methods.

Name of Specimen	Injection-Mold Condition	Methods of Surface Treatment
PP-Ref	Non-etched mold	None
PPm-Ref	Etched mold	
PP-Pl	Non-etched mold	Plasma treatment
PPm-Pl	Etched mold	
PP-Pr	Non-etched mold	Primer coating
PPm-Pr	Etched mold	
PP-Fl	Non-etched mold	Flame treatment
PPm-Fl	Etched mold	

**Table 2:** Surface roughness parameters of PP specimens.

Name of Specimen	Rpv (μm)	Rq (μm)	Ra (μm)	Rz (μm)	Rsk	Rku
PP-Ref	0.586 ± 0.332	0.053 ± 0.004	0.056 ± 0.023	0.362 ± 0.012	−0.170 ± 0.031	5.618 ± 0.012
PP-Pl	0.938 ± 0.255	0.121 ± 0.017	0.133 ± 0.060	0.776 ± 0.158	0.025 ± 0.112	2.565 ± 0.243
PP-Pr	0.307 ± 0.158	0.026 ± 0.005	0.030 ± 0.019	0.096 ± 0.098	−0.021 ± 0.072	3.528 ± 1.019
PP-Fl	0.653 ± 0.040	0.084 ± 0.006	0.062 ± 0.007	0.641 ± 0.044	0.223 ± 0.567	4.034 ± 0.011
PPm-Ref	2.824 ± 0.685	0.723 ± 0.220	0.407 ± 0.135	3.676 ± 0.985	−0.193 ± 0.078	2.409 ± 0.035
PPm-Pl	3.467 ± 0.786	0.634 ± 0.108	0.437 ± 0.148	3.741 ± 0.619	0.040 ± 0.003	2.827 ± 0.221
PPm-Pr	1.482 ± 0.345	0.161 ± 0.003	0.146 ± 0.006	1.264 ± 0.003	0.381 ± 0.113	5.594 ± 0.365
PPm-Fl	2.754 ± 0.804	0.445 ± 0.219	0.352 ± 0.128	2.493 ± 0.957	−0.012 ± 0.291	2.504 ± 0.121

## Data Availability

Not applicable.
